# Properties of Residue from Olive Oil Extraction as a Raw Material for Sustainable Construction Materials. Part I: Physical Properties

**DOI:** 10.3390/ma10020100

**Published:** 2017-01-25

**Authors:** Almudena Díaz-García, Carmen Martínez-García, Teresa Cotes-Palomino

**Affiliations:** Department of Chemical, Environmental and Material Engineering. Higher Polytechnic School of Linares, University of Jaen, Scientific and Technological Campus of Linares, 23700 Linares (Jaén), Spain; almudenadiazgarcia@gmail.com (A.D.-G.); mtcotes@ujaen.es (T.C.-P.)

**Keywords:** wet pomace, sustainable construction materials, circular economy

## Abstract

Action on climate, the environment, and the efficient use of raw materials and resources are important challenges facing our society. Against this backdrop, the construction industry must adapt to new trends and environmentally sustainable construction systems, thus requiring lines of research aimed at keeping energy consumption in new buildings as low as possible. One of the main goals of this research is to efficiently contribute to reducing the amount of residue from olive oil extraction using a two-phase method. This can be achieved by producing alternative structural materials to be used in the construction industry by means of a circular economy. The technical feasibility of adding said residue to ceramic paste was proven by analyzing the changes produced in the physical properties of the paste, which were then compared to the properties of the reference materials manufactured with clay without residue. Results obtained show that the heating value of wet pomace can contribute to the thermal needs of the sintering process, contributing 30% of energy in pieces containing 3% of said material. Likewise, adding larger amounts of wet pomace to the clay body causes a significant decrease in bulk density values.

## 1. Introduction

Human beings spend more than 90% of their lives in built environments, where noise and extreme temperatures are unwanted elements that affect people’s rest and everyday activities [1]. According to research, more than 75% of buildings have serious deficiencies in their acoustic and thermal insulation capacity, meaning it is necessary to add greater acoustic and thermal insulation features to the materials currently used in the construction process [2]. The building envelope is responsible for the need for air conditioning in buildings, which in housing represents approximately 50% of the total energy consumption [3]. One of the most important strategies aimed at achieving good energy efficiency in buildings involves improving the quality of building envelopes by using materials with lower thermal conductivity to improve insulation capacity [4].

Ultimately, technological and innovative development is an urgent need in the construction sector, which means this industry, needs to adapt to the latest trends and to new environmentally sustainable construction systems. This requires both new materials with higher insulation features, as well as construction solutions specially aimed at refurbishment [5]. Until recent times, the development of materials and products aimed at satisfying new building trends did not play a relevant role in the construction materials industry.

It is also important to stress that new European regulations strongly favor the use of materials offering better thermal and acoustic characteristics. This has had a great impact on the sector, given that only bricks with high-density parameters are permitted, meaning an increase in thickness parameters in order to obtain optimal insulation values. It is therefore urgent that the structural ceramic sector incorporates acoustic and thermal insulating criteria when designing and manufacturing new products. The insulation and acoustic capacity of ceramic material will depend on the balance between porosity and elasticity modulus, although pore size, geometry, and orientation are also important. In consideration of the fact that the thermal and acoustic insulating capacity of a material therefore depends largely on its porosity, the development of materials with controlled porosity has become an area of great interest for the construction industry [5].

Materials frequently used as pore-forming agents for production of ceramic bricks can be classified as organic or inorganic. Sawdust, polystyrene, paper sludge, coal, coke, and petroleum are examples of pore-forming organic materials, while pearlite, calcite, diatoms, and vermiculite are inorganic pore-forming agents. Organic materials are generally less expensive than inorganic materials and also feature the advantage of contributing heat to the ceramic firing process, while the main drawback of these materials is the emission of CO_2_. Inorganic pore-forming materials offer fewer environmental problems but can change the plasticity of clay in a negative way and increase the need for water to maintain clay in optimal conditions.

Organic residues are widely used as pore-forming agents in the ceramic industry [6,7,8,9]. For many years now, construction materials have been recipients of residues from other industrial sectors, for example: petrochemical residues, sewage sludge ash from MSW (municipal solid waste), residues from the paper and beer industries, agricultural residues, among others. [10,11]. The use of residues offers a number of advantages: the optimization of the properties of the ceramic products, as well as lower costs and a solution to the environmental problem caused by disposing of the residues in landfill sites. Furthermore, their use could help obtain green building certifications, given that these certifications favor products containing 10%–20% of recycled material [12,13], and would also help obtain the certification in the European Union Ecolabel environmental labeling system (Regulation (EC) No. 66/2010 of the European Parliament and of the Council of 25 November 2009 on the EU Ecolabel). In this respect, the ceramic and cement industries feature manufacturing processes that offer a viable valorization of organic and inorganic residues [14,15]. This valorization can be obtained by using the heating value from the combustion process and/or by adding the residue to the inner structure of the materials so that it becomes part of its matrix and, therefore, an inert element [14,15,16,17,18,19,20,21,22,23,24,25,26].

The goal of this research is to apply the principles of the circular economy by evaluating the use of waste products derived from olive oil production as a raw material in the production of structural ceramic materials. The sample properties of the ceramic are then tested in order to be compared with materials made without the added waste.

## 2. Materials and Methods

### 2.1. Raw Materials

**Figure 1:**
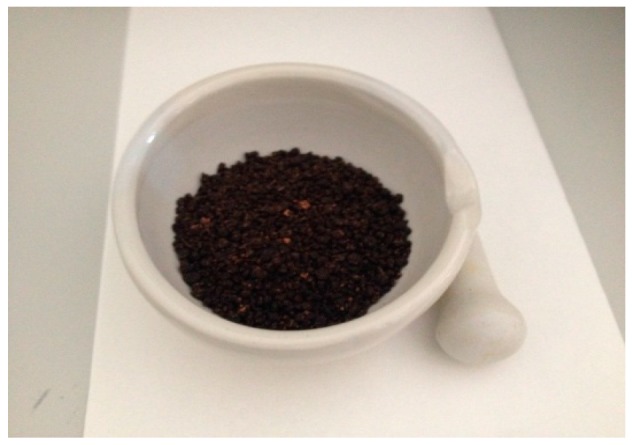
Wet pomace.

The clay used was supplied by a clay-pit located in Bailén (Spain) and was obtained by mixing different percentages of three types of clay: 40% black, 30% yellow, and 30% red clay. The wet pomace was collected from a local olive oil production plant using the two-phase method (Figure 1). The composition of wet pomace depends on several factors [27]. The waste was dried in an oven at 90 °C for 24 h until dry. 

The clay and olive pomace was then ground in a hammer mill using a 0.5 mm sieve. The total content of carbon, hydrogen, nitrogen, and sulfur of the dry residue was determined by combustion of samples in an O_2_ atmosphere using a CHNS-O and a Thermo Finnigan Elementary Analyzer Flash EA 1112. The technical analysis of the wet pomace was carried out using a thermal analyzer TGA/DSC 1 by METTLER TOLEDO. The module is made up of a HT1600 horizontal furnace that works between room temperature (RT) and 1600 °C, and an MX5 ultra-microbalance connected to a DSC HSS2 model Pt-Rh sensor. The balance was thermostatted by means of a bath at 22 °C and protected with a continuous flow of N_2_ at a rate of 20 mL/min.

**Figure 2:**
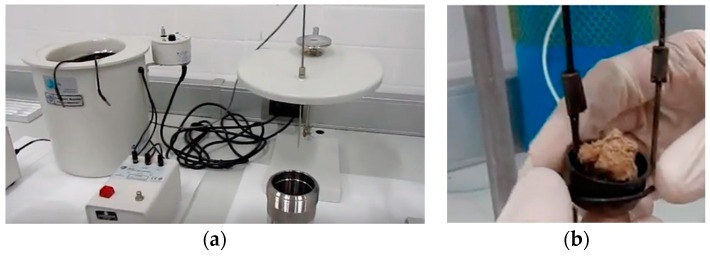
(**a**) Oxygen bomb calorimeter; (**b**) Sample in bomb calorimeter.

The qualitative determination of the main crystalline mineralogical phases present in clay and wet pomace was carried out using X-ray diffraction (XRD). This technique was used because each X-ray diffractogram is characteristic of the material analyzed [28]. The higher heating value (HHV) of wet pomace was determined using a Parr 1341 Plain Oxygen Bomb Calorimeter (Figure 2), observing the UNE 32006:1995 standard [29,30].

### 2.2. Preparation Process

The cold forming process prior to sintering the test pieces, whose approximate dimensions were 117 × 28 × 17 mm, was carried out by extrusion (Verdés, Monoboc 050-C/OR). The vacuum levels were variable depending on the porosity level desired, reaching maximum values of 85%–90% and a pressure of 7–8 bars in the extrusion press.

Once the test pieces were formed, it was necessary to eliminate most of the water before moving on to the sintering phase as, otherwise, water would be expelled violently, resulting in fissures and affecting the test piece structure. Given that drying is a delicate operation that must be strictly controlled, the formed pieces were introduced in a drying furnace at 110 °C for 48 h before undergoing the sintering process.

The firing treatment for the formed test pieces was carried out at 3 different temperatures (850, 950, and 1050 °C) for each wet pomace percentage, in a TECNO PIRO electric laboratory furnace model B4-A. The sintering ramp used consisted of the following stages: (1) from ambient temperature to 400 °C: the speed of warming in the oven is 4 °C/min; (2) from 400 °C to 700 °C: the speed of warming in the oven is 2 °C/min; (3) from 700 °C to final temperature: the oven heating rate is 1 °C/min; (4) maintenance of the maximum temperature for 3 h; (5) cooling by natural convection to ambient temperature.

### 2.3. Sintered Materials

Once the pieces were sintered, the following physical properties were determined:

Weight loss: for this test, 10 pieces were chosen from each clay-wet pomace mixture percentage to be exposed to each of the 3 temperatures, making a total of 30 pieces for each mixture percentage, 120 pieces in total. After undergoing the sintering process, the weight loss of the test pieces was calculated by subtracting the weight of each piece before the firing process (but after furnace drying) from the weight of each piece after firing. The laboratory balance used to do this test has a precision of ±0.001 g.

Water absorption test by immersion in cold water: absorption is a way of measuring moisture penetration when a brick is fully submerged in water during a long period of time. This test was carried out in accordance with standard UNE 772-21 dated September 2011 [31].

Compressive strength study: this test was carried out following standard UNE-EN 772-1 dated September 2011 [32]. This European standard specifies the method to be used to determine compressive strength of pieces made in a masonry factory. The minimum number of test pieces must be six. 

Before beginning the study, the test pieces underwent prior conditioning, by which they were dried in a ventilated furnace at 110 °C until they reached a constant mass. Constant mass is reached if the pieces are weighed two consecutive times in a 24 h interval during the drying process and the mass loss difference between the two times is not greater than 0.2% of the total mass. They were then left to dry until room temperature was reached.

Modulus of rupture (MOR) was obtained from a three-point bending strength test (HOYTOM, CMC, 100 mm Span using a load cell of 5 kN, 100 mm span, and a displacement of 5 mm/min. Reported results are the mean value and standard deviation of six determinations.

**Figure 3:**
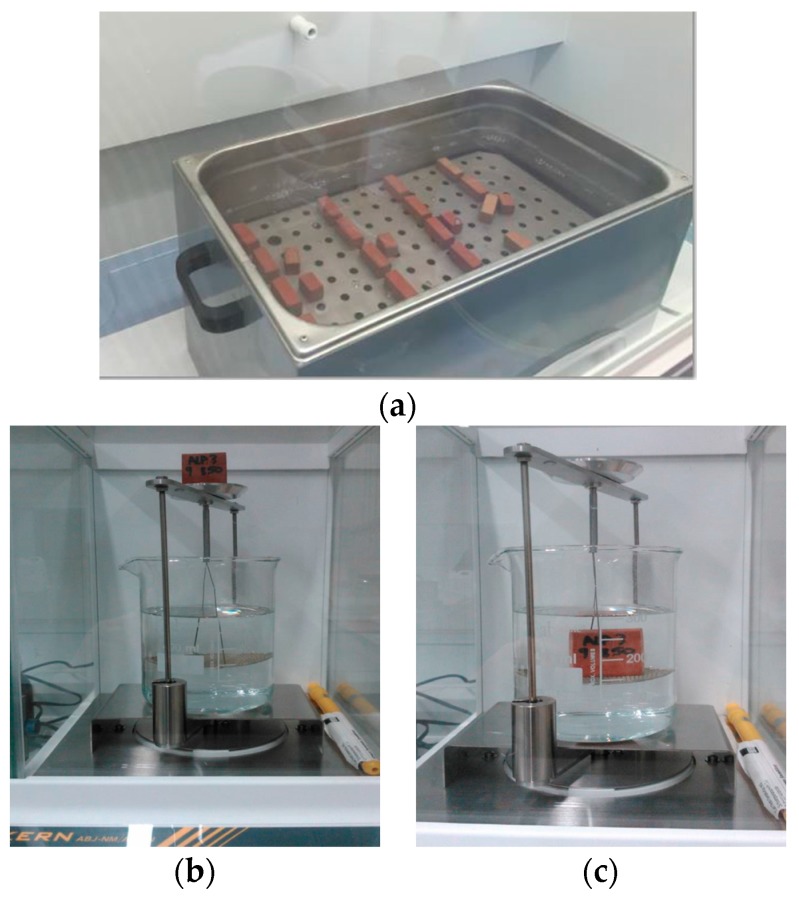
Apparent porosity and bulk density test. (**a**) Saturation in water; (**b**) Determination suspended mass; (**c**) Determination saturated mass.

The apparent porosity and the bulk density were measured according to ASTM C373 (Figure 3), which involves drying the test specimens to constant mass (D). After impregnation, the mass (S) while suspended in water and saturated mass (M) of each specimen were determined. The apparent porosity, P(%), expresses the relationship of the volume of open pores with the exterior volume of the specimen and is calculated as follows: P = [(M − D)/(ρ × V)] × 100, where V (cm^3^) is the exterior volume (V = M − S) and ρ is the density of the water, 1 g·cm^−3^. The bulk density, B (g·cm^−3^), of a specimen is the quotient of its dry mass divided by the exterior volume, including pores: B = D/V [33].

Freezing-thawing test: this study was carried out following the UNE-EN 67028 standard [34]. The goal of this standard is to describe the test method to be used to determine the behavior of construction bricks when exposed to ice. The sample size was 6 test pieces, and any visible structural defects appearing in each one were registered.

Leaching test: the leaching test was carried out to determine the risk of the test pieces leaching. The test used was toxicity characteristic leaching procedure (TCLP), which in turn is based on the DIN 38414-S2 standard [35].

## 3. Results

### 3.1. Raw Materials

Table 1 shows the results obtained by the measuring team for the three types of clay and the wet pomace.

**Figure 4:**
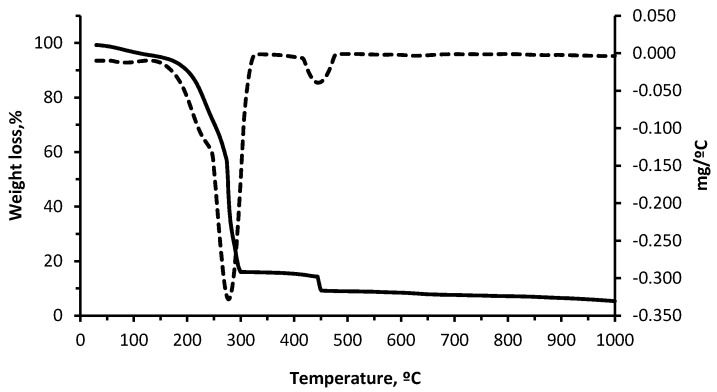
Thermogravimetric/differential thermogravimetric (TG-DTG) curves of wet pomace.

**Figure 5:**
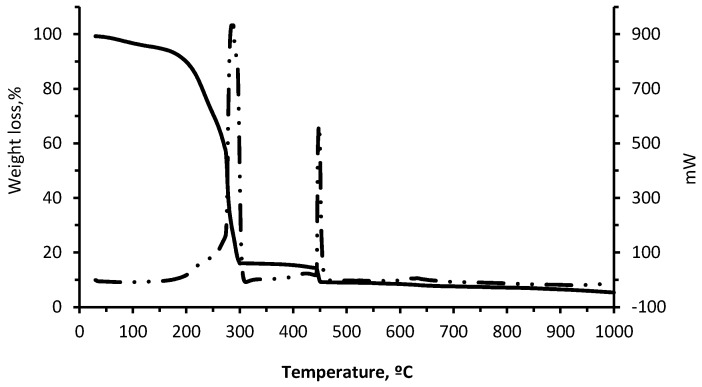
Thermogravimetric/differential scanning calorimetry (TG-DSC) curves of wet pomace.

The thermal analysis for the residue was carried out by obtaining thermogravimetric/differential thermogravimetric (TG-DTG) and TG/differential scanning calorimetry (TG-DSC) curves (Figure 4 and Figure 5), both based on the temperature for the material. Figure 4 shows the results of the thermal analysis while submitting the residue to a thermal cycle of up to 1000 °C.

**Figure 6:**
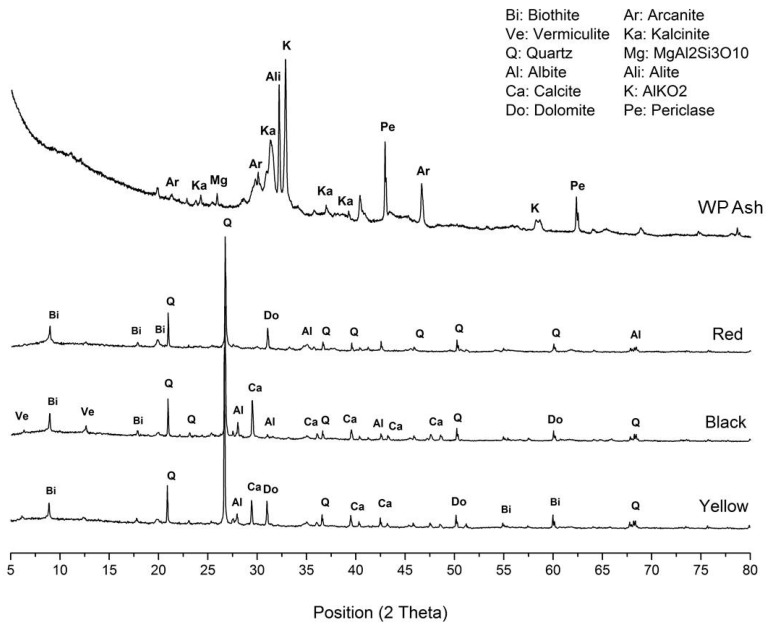
Wet pomace and clays diffractograms.

The analysis of the mineralogical phases of the different types of clay and wet pomace used to make the pieces is shown in Figure 6.

Table 2 shows the results of the higher and lower heating value analysis for the wet pomace.

### 3.2. Sintered Material

The test pieces formed were classified according to codes ALP 0, ALP 3, ALP 7, and ALP 10 according to the percentage of residue added to the extruded mixture. Each test piece was marked with the corresponding code and assignation number.

**Figure 7:**
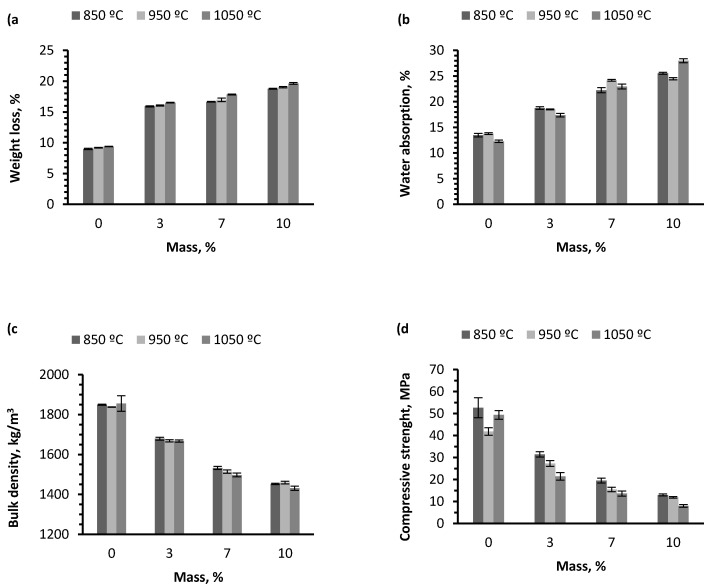
Physical properties of samples with wet pomace dosage from 0% to 10%: (**a**) Weight loss; (**b**) Water absorption; (**c**) Bulk density; (**d**) Compressive strength.

Figure 7 shows the physical properties of material sintered: weight loss, water absorption, bulk density, and compressive strength.

Table 3 shows results obtained for modulus of rupture (MOR) for the bending strength test.

Table 4 shows data obtained for the open porosity of the pieces.

Finally, the leaching test was carried out for samples sintered at 850 °C and containing the maximum percentage of residue, namely 10%. Results are shown in Table 5.

## 4. Discussion

Table 1 shows that the organic matter content of the different types of clay is low, but superior to the nitrogen and hydrogen content, which concurs with the characteristic composition of natural clay found in existing literature. The organic matter content (generally humus) is highly important due to its influence on the colloidal properties, the firing, and the color [36]. As can be seen in the table, the carbon, nitrogen, and hydrogen content in the residue is high, due to the fact that it is vegetal organic matter. Furthermore, it was proven that the elemental percentages are similar to the percentages found in the elemental analysis results contained in the literature for the same kind of residue and for other types of residues such as bagasse from the brewing industry [9]. It can therefore be assumed that it will have a high heating value.

The TG-DTG and TG-DSC curves for wet pomace (Figure 4 and Figure 5) are typical of solid combustible matter. The first weight loss decrease (5%) observed between 25 and 150 °C is caused by the physical evaporation of the water contained in the material, while the second weight loss (77.75%) observed in the interval between 150 °C and 300 °C may be due to the combustion of volatile organic compounds (VOCs). The third and last weight loss (6.95%), which coincides with the second exothermic peak at 450 °C, is due to the combustion of fixed carbon.

It can be observed that the residue can be totally combusted in the clay body at low temperatures. According to the DSC curve, there are two peaks corresponding to the two exothermic reactions in the 150–500 °C interval, with maximums of 300 °C and 450 °C corresponding to the combustion of the VOCs and the respective fixed carbon. The total weight loss is 90% at approximately 1000 °C, which indicates that very little ash is produced during combustion. Exothermic reactions of wet pomace positively contribute to the energy needs of the sintering phase for the ceramic pieces containing wet pomace [9,27,37].

From a global point of view, the diffraction patterns of the three types of raw clay indicate that they contain mainly quartz (SiO_2_), calcite (CaCO_3_), biotite (iron and aluminum phyllosilicate), and dolomite (CaMg(CO_3_)_2_) and, to a lesser degree, other phyllosilicates such as vermiculite, as well as plagioclases like albite (Figure 6). A greater presence of calcium and magnesium carbonate in the clay influences the subsequent thermal treatment because these are broken down, yielding the corresponding oxide and releasing CO_2_. The temperature at which this breakdown occurs varies between 450 °C for magnesite and 950 °C for calcite. The release of CO_2_ must be controlled to prevent it from happening in a violent manner [36].

The analysis of the crystalline phases for wet pomace was carried out using a sample of ash taken from its combustion in a laboratory furnace. It is worthwhile to emphasize the high content of non-diffracting material in the ash. In the wet pomace sample, the crystalline component present to a greater degree is calcite, followed by aluminates and potassium silicates, magnesium and aluminum silicates and aluminum, as well as some halite. As this was a partial identification, it would be necessary to analyze the X-ray fluorescence data to reliably determine the crystalline phases present.

According to the results of Table 2, it can be said that an important proportion of the thermal energy needed to produce the ceramic pieces comes from the combustion of the residue being studied and, consequently, it is a way of saving energy because less electricity will be required to operate the furnace. During thermal treatment, weight and color changes were observed in test samples. According to the findings, it can be stated that adding incremental quantities of wet pomace results in an increase of weight loss due to calcination (Figure 7a). This occurs because the residue has a high content of organic matter, which is consistent with elemental and heating value analyses.

By analyzing the data shown in Figure 7b, a positive trend in water absorption can be expected in the pieces as the residue percentage increases. This is due to the fact that the greater the amount of residue added to the ceramic mixture, the higher the porosity percentage formed in the matrix. The water absorption percentage increases considerably in pieces that contain 10% wet pomace.

On the other hand, data deviations obtained for each group at the three sintering temperatures were not very widespread; it can therefore be affirmed that the firing temperature did not affect the water absorption capacity of the ceramic pieces. This physical property considerably affects the final quality of the material, as well as its durability. High wet pomace content can therefore produce defects and a tendency to absorb water, which results in less durability of the ceramic materials [28].

Figure 7d shows how the sintering temperature of the pieces does not have a significant effect on their compressive strength. This behavior can be observed in the three different percentages of residue addition. Samples sintered at 850 °C exhibit greater compressive strength when compared to samples fired at 950 °C and at 1050 °C for the three percentages of residue addition. Alternatively, the increased percentage of residue added to the ceramic mixture produces a decline in the compressive strength values. A higher compressive strength is therefore observed in test pieces containing 3% wet pomace, followed by pieces with 7% and, finally, pieces with 10%. The reason for this behavior is that a greater number of pores are formed in the sample containing 10% wet pomace, which causes density to be lower than in other samples and weakens the inner structure of the test piece. Pieces that display higher compressive strength are those containing 3% wet pomace and sintered at 850 °C, while test pieces with lower compressive strength are those containing 10% wet pomace and sintered at 1050 °C. The difference between both is 23.42 MPa, which implies a significant difference regarding the construction features of both types of pieces.

The results obtained in the bending strength test shown in Table 3 are in accordance with the values obtained in the compressive strength test, where the samples with a higher content of wet pomace present a modulus of rupture minor to the pieces with a lower percentage waste added because of great porosity generated for a higher organic content in these samples. This trend increases at elevated temperatures due to the open porosity in the series sintered at 1050 °C being more elevated than the series sintered at 850 and 950 °C. An increase in open porosity causes a greater fragility of the pieces.

Figure 7c shows the bulk density test results. As can be expected, the trend in the series of samples is a decrease in bulk density as the percentage of residue in the samples increases, due to greater loss of organic matter in firing combustion, which implies an increase of total porosity of the material. The bulk density varies, starting at the higher value of 1855 kg/m^3^ for pieces containing 0%, until the lower value of 1431 kg/m^3^ for pieces coded ALP10, both sintered at 1050 °C. This represents a bulk density reduction of 22.86% in pieces at this temperature. At the three sintering temperatures tested, results show bulk density values that are very close numerically in samples containing a certain percentage of residue weight. It can therefore be concluded that the firing temperature does not have a significant effect on the final bulk density of the pieces.

The open porosity results of Table 4 show that the quantity of residue added to the mixture is directly proportional to the quantity of pores obtained in the ceramic material. If ceramic pieces contain greater quantities of wet pomace, the open porosity in their matrix increases, reaching the maximum value of 46% in the samples coded ALP10 and sintered at 1050 °C. As expected, the lowest level of open porosity is found in pieces without residue. As they do not have an alternative source of organic matter provided by the residue, the level of porosity formed in the interior is not greater than that derived from the composition of the mixture of clays. The greater increase of open porosity is found in the series sintered at 1050 °C, with an increase of about 67% with regard to the initial value.

With regard to the effect of temperature on this property, a slight increase in porosity was observed in pieces containing the same percentage of residue but treated at a higher firing temperature. Each sintering temperature increased open porosity of the pieces by approximately 1%–2% when compared to the previous temperature.

**Figure 8:**
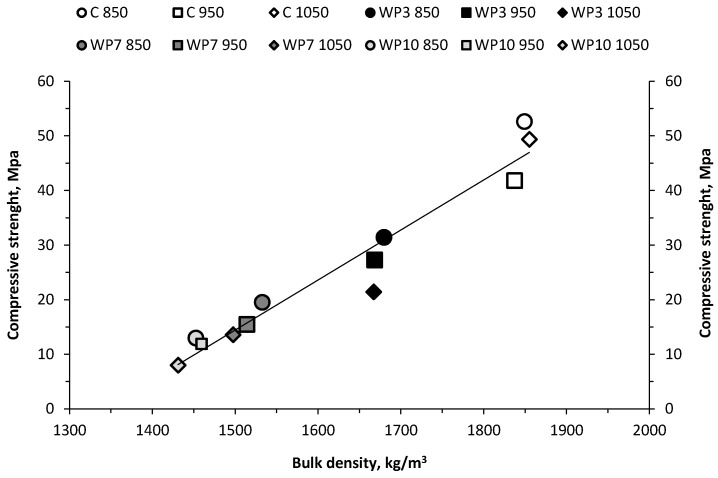
Relationship between bulk density and compressive strength of wet pomace bricks.

The relationship between bulk density and compressive strength is analyzed in Figure 8, which shows how the relationship between the two properties is directly proportional: as the density of the pieces increases, compressive strength rises too, while both properties prove to be inversely proportional to the quantity of residue added [38]. Hence, as the percentage of added residue increases, there is a greater weight loss on account of the organic matter combustion in the residue, which leads to a decrease in bulk density and an increase in open porosity that weakens the inner structure and reduces the compressive strength. This trend can be observed in the three temperatures being studied.

A numeric model proposed by Aouba et al. (2016) [39] was tested in order to predict compressive strength of clay samples that incorporate organic residue. This method proposes the calculation of the parameter “a” to determine the adjustment between the theoretical data of the model and the experimental data. This calculation was made due to the importance of mechanical properties in the investigated samples, given that they are to be used as construction materials. The development of numeric models to predict the compressive strength of clay bricks should also be of interest to manufacturers, who can then study the possibility of using waste while meeting the required specifications of their products. This is a normalized function that associates compressive strength of each sample with the percentage of organic matter added.

(1) $$\sigma_{cx}=\frac{\sigma_{c0}}{1+\frac{x}{a}}$$

where:

σ*_cx_*, compressive strength of the material that adds x% organic residue
σ*_c_*_0_, compressive strength of the material that adds 0% organic residue
*x*, % of organic residue added
*a*, parameter that depends on the type of organic matter

**Figure 9:**
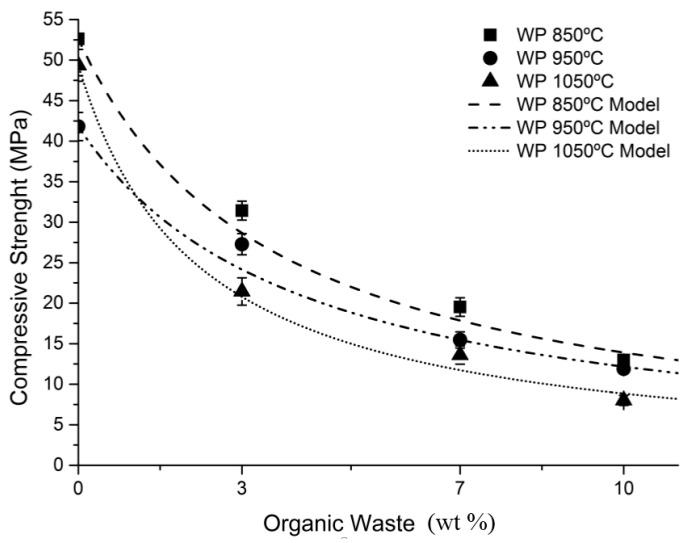
Relationship between compressive strength and wt % of organic matter.

Figure 9 shows the results of the predictive analysis. Linear regression with the method of least squares was used to determine the function giving the normalized mean compressive strength for each sample according to the amount of incorporated wet pomace. The results describe how compressive strength evolves with the percentage of wet pomace added at the three working temperatures. Table 6 shows the statistical analysis of the regression performed, which shows a high level of coincidences between the prediction Equation (1), and the experimental data obtained in the 0–10 wt % interval of wet pomace added at the tested temperatures.

The results obtained in the bending strength test according to values obtained from the compressive strength test show that the pieces with a higher content of wet pomace present a modulus of rupture minor to that of pieces with a lower percentage added, which is due to the increased porosity generated by a higher organic content in these pieces. This trend increases at higher temperatures due to the open porosity in the series sintered at 1050 °C being more elevated than series sintered at 850 and 950 °C. The increased open porosity causes the pieces to be more fragile.

**Figure 10:**
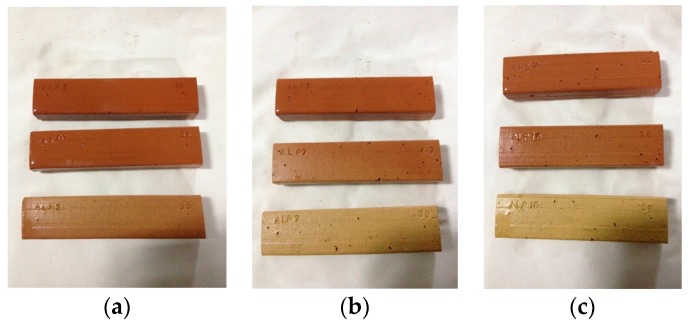
Pieces ALP 3% (**a**), 7% (**b**), and 10% (**c**) at 850 °C, 950 °C, and 1050 °C after the 25th freezing- thawing cycle.

The results obtained in the freeze-thaw test are satisfactory since none of the pieces suffered any damage after the 25 freezing-thawing cycles. However, some of the pieces containing a greater percentage of waste (ALP 10%) suffered superficial flaking. Figure 10 shows the effects of the freezing-thawing stages undergone by the pieces.

Finally, the leaching test was carried out for samples sintered at 850 °C and containing the maximum percentage of residue, namely 10%. Results are shown in Table 5. Having compared the values obtained from the elements analyzed using the inductively couple plasma (ICP)-mass technique, with the maximum values allowed for the TCLP test, it was observed that none of the elements exceeded the established limits and, therefore, it can be concluded that the ceramic pieces do not generate any kind of leaching that is toxic for the environment.

## 5. Conclusions

From the results obtained in this study, it was determined that bricks produced with this type of waste obtained from olive oil production by means of the two-phase extraction process indicate the potential to manufacture high-quality materials. This possibility is beneficial not only from an economic point of view, as it can lead to savings for companies when buying raw material, but also from an environmental perspective, given that it entails the elimination of waste that is currently an unresolved problem and leads to a damaging, physical-chemical impact on the environment, a situation that needs to be solved in an efficient manner. The experimental results obtained during this research allow us to establish that the material characterization carried out can be used to optimize construction features and benefits of the fired clay ceramic pieces.

In particular, the following conclusions are proposed:

First, adding wet pomace to the clay test pieces that were made for the purposes of the tests did not cause any problems during the extrusion, drying, and sintering phases of the ceramic pieces. The proportion of residue is a key factor that affects the technological properties of the bricks. It was proven that water absorption increases if the percentage of residue added to the clay is increased and the firing temperature is higher; for example, there is a 15% water absorption increase in the pieces containing 10% wet pomace when compared to pieces with 0% of residue added.

Adding incremental quantities of wet pomace to the clay body produces significant reductions in the bulk density and increases open porosity, which can lead to changes in the properties, such as thermal conductivity, which determines the thermal insulation capacity of the materials. With regard to the results obtained from the mechanical property studied, it was established that compressive strength is reduced when the proportion of wet pomace in the ceramic pieces is increased. However, only the values for the pieces sintered at 1050 °C and containing 10% wet pomace fail to comply with the AENOR standard. Considering higher compressive strength specifications and a lower sintering temperature, which result in the need for less working energy in the furnace, it can be concluded that the pieces with 3 wt % wet pomace and sintered at 850 °C can be considered the best quality bricks. However, pieces with residue values of 7% and 10%, and sintered at 850 °C, showed a compressive strength value higher than the limit value required by the certification body.

On the other hand, implementing the use of numeric models for predicting compressive strength can be a very useful tool for manufacturers in order to comply with the specifications established for the materials used. However, the results shown must be deemed as specific for wet pomace. The results obtained in the leaching test performed on the pieces shows that there is no risk that the ceramic pieces might produce elements that are toxic to the environment.

Lastly, it was proven in the section on determination of the heating value of wet pomace that, due to the high combustion heat produced, adding this residue to the ceramic matrix can contribute to the thermal needs of the sintering process. As a consequence of the energy provided during the firing process of the ceramic pieces, electricity consumption is reduced in proportion to the amount of thermal energy produced.

Ultimately, this experimental work concludes by stating that the incorporation of an organic residue to the manufacturing process of ceramic materials positively contributes to the preservation of the environment by reducing CO_2_ emissions in the atmosphere and eliminating a potentially toxic residue. Furthermore, the high heating value of the wet pomace also helps reduce the monetary cost of the sintering phase.

## Figures and Tables

**Table 1:** Elementary composition of waste and clays.

	% N	% C	% H
Yellow clay	0.0395 ± 0.0004	1.8906 ± 0.0406	0.3205 ± 0.0240
Red clay	0.0448 ± 0.0005	1.0356 ± 0.0321	0.4591 ± 0.0264
Black clay	0.0780 ± 0.0028	3.3472 ± 0.1418	0.3331 ± 0.0039
Wet pomace	1.5944 ± 0.0439	48.2627 ± 0.2066	7.2396 ± 0.0018

**Table 2:** Higher heating value (HHV) and lower heating value (LHV) of the wet pomace.

Waste	HHV, kJ/kg	LHV, kJ/kg
Wet pomace	18,860.16 ± 34.85	19,706.74 ± 73.05

**Table 3:** Modulus of rupture (MOR) (MPa) for bending strength test of samples.

Firing Temperature	850 °C	MOR, MPa 950 °C	1050 °C
ALP 3	12.4 ± 0.4	9.8 ± 0.3	8.9 ± 0.1
ALP 7	7.6 ± 0.1	6.8 ± 0.1	5.4 ± 0.7
ALP 10	6.0 ± 0.4	5.8 ± 0.2	4.3 ± 0.7

**Table 4:** Open porosity (%) of samples.

Firing Temperature	850 °C	950 °C	1050 °C
ALP 0	28.99 ± 0.26	29.15 ± 0.26	27.55 ± 0.24
ALP 3	33.96 ± 0.76	35.22 ± 0.40	36.15 ± 0.33
ALP 7	39.53 ± 0.47	41.58 ± 0.33	42.66 ± 0.46
ALP 10	43.24 ± 0.11	44.52 ± 0.19	46.09 ± 0.31

**Table 5:** Leaching test results.

Element	Maximum ppb Permitted in the TCLP Test	Sample Concentration, ppb
^53^Cr	5000	363.382 ± 2.097
^60^Ni	400–2000	5.580 ± 0.197
^63^Cu	2000–10,000	50.478 ± 0.520
^66^Zn	2000–10,000	26.308 ± 0.591
^75^As	200–1000	20.633 ± 0.705
^82^Se	1000	16.066 ± 0.045
^107^Ag	5000	0.183 ± 0.172
^111^Cd	500–100	0.138 ± 0.062
^208^Pb	400–2000	2.506 ± 0.259

TCLP: Toxicity characteristic leaching procedure.

**Table 6:** Coefficient a, coefficient of correlation, and standard error.

	Coefficient of Determination, r^2^	a	
		Value	Std Error
ALP 850 °C	0.960	3.593	0.268
ALP 950 °C	0.983	4.106	0.220
ALP 1050 °C	0.989	2.183	0.172
